# The detection of capsaicin and dihydrocapsaicin in horse serum following long-term local administration

**DOI:** 10.1186/s12917-018-1518-9

**Published:** 2018-06-19

**Authors:** A. Zak, N. Siwinska, M. Slowikowska, H. Borowicz, P. Szpot, M. Zawadzki, A. Niedzwiedz

**Affiliations:** 1 Department of Internal Medicine and Clinic of Diseases of Horses, Dogs and Cats, Faculty of Veterinary Medicine, Wroclaw University of Environmental and Life Sciences, pl. Grunwaldzki 47, 50-366 Wroclaw, Poland; 2 Department of Forensic Medicine, Wroclaw Medical University, ul. Mikulicza-Radeckiego 4, 50-345 Wroclaw, Poland

## Abstract

**Background:**

Capsaicin and dihydrocapsaicin are alkaloids with analgesic effects in humans and animals. When used locally, both of them minimalise pain sensation by defunctionalising nerve endings. According to the Federation Equestrian International Prohibited Substances List, these are substance banned in horse competitions. The aim of the study was to determine the detection time of capsaicin in both plasma and serum after long-term use of a gel recommended for commercial use and applied as intended. The objective of the study was to select the best material for the detection of capsaicin as a doping substance in horses.

**Methods:**

Nine healthy mature horses were administered 0.1% capsaicin topically in the form of a commercial analgesic gel (15 g of the gel per limb) to the front limbs every 24 hours for five days with a polar fleece bandage. Blood serum and plasma were collected prior to gel application and in the 12th, 18th, 24th, 36th, 42nd, 48th, 60th, 84th, 108th, 132nd, 156th hour after the gel application. Qualitative and quantitative analysis was performed using ultra-high performance liquid chromatography coupled with a triple quadrupole mass spectrometry (UHPLC-QqQ-MS/MS).

**Results:**

The concentration of capsaicin in the serum samples did not exceed the lower limit of quantification. Capsaicin was not detected in the plasma samples during the entire study period. Dihydrocapsaicin was not detected in blood serum or plasma.

**Conclusion:**

The presented results suggest that capsaicin is not detected in horse serum in the 24-hour-periodfollowing its last application according to the dosage regimen used by owners and veterinarians for therapy rather than doping, based on a five day gel application and a polar bandage.

## Background

Capsaicin (8-Methyl-N-vanillyl-trans-6-nonenamide) is an alkaloid organic compound from the *Capsicum* family [1]. Large amounts of capsaicin are found in various peppers and give them a sharp taste [2]. Capsaicin is commonly used by humans locally due to its analgesic properties, mainly in the treatment of back pain, neurogenic pain, osteoarthritis and postherpetic neuralgia [2]. It has gastroprotective properties when administered orally and minimalises postoperative nausea and vomiting when used in acupressure [2]. In horses, it is mainly included in analgesic and anti-inflammatory heat rub creams used in lameness, laminitis and arthritis [3]. In a reversible model of equine foot lameness, the degree of lameness and other parameters indicating pain sensation significantly decreased in horses treated topically with capsaicin from 40 to 240 min after its application [3]. Capsaicin is also used as a wood chewing deterrent [4]. Dihydrocapsaicin (N-(4-Hydroxy-3-methoxybenzyl)-8-methylnonanamide) is a capsaicinoid analog found in spicy peppers. It is a capsaicin derivative that is an irritant but also has analgesic properties. Hence, it is also used in analgesic creams [5].

Capsaicin is included in the 2017 Equine Prohibited Substances List created by the Federation Equestrian International (FEI). Hence, it should be identified easily and rapidly in horses [6]. Ultra-high – performance liquid chromatography – tandem mass spectrometry (UHPLC- MS-MS) is the newest method for the detection of capsaicin in plasma. Due to its selectivity, sensitivity, repeatability and time of detection, it is considered the best method for the rapid analysis of capsaicin [7]. The aim of the study was to determine the time of capsaicin detection in the blood following its topical administration in the form of a gel with the dosage regimen based on the most common guidelines. The second objective was to compare capsaicin detectability in blood serum and plasma and to choose the best diagnostic material.

## Methods

All experimental procedures were performed with the approval of the Animal Experimentation Local Ethics Committee in Wroclaw (permit No 97/2016). Nine horses (seven mares and two geldings) with a mean age of 13.7 years (range: 6–18 yo), a mean weight of 436.1 kg (range: 300–517 kg) and of various breeds were included in the study. These horses were nonworking and were kept at the Department of Internal Medicine and Clinic of Diseases of Horses, Dogs and Cats of the Wroclaw University of Environmental and Life Sciences for didactic student training. The horses were maintained in a stable-pasture system with full-time access to hay, water and mineral licks and were fed oats based on their nutrient requirements, body weight and level of physical activity. Prior to inclusion in the study, the horses underwent clinical and orthopaedic examinations, which were unremarkable. Prior to commencing the study, 10 mL of venous blood was collected from each horse at T0 into tubes^1^ containing ethylenediaminetetraacetic acid (EDTA) as an anticoagulant and plain tubes^1^ using an 18G needle. On the first day of the study, 15 g (weighed using a scale^2^) of a commercial gel containing 0.1% capsaicin^3^ was applied to each thoracic limb from the level of the carpal joint to the pastern joint at 8 pm. Latex gloves were worn during the application of the capsaicin gel. The gel was massaged into the limb for five minutes. Then, a polar fleece bandage was placed on the digit for 12 h. The application procedure was repeated every 24 h for five days. Venous blood was collected from the jugular vein prior to the application of the gel (T0), then 12 (T12), 18 (T18), 24 (T24), 36 (T36), 42 (T42), 48 (T48), 60 (T60), 84 (T84), 108 (T108), 132 (T132), 156 (T156) hours following the last gel application. The samples were allowed to settle on ice for 30 min. The plasma (5 min, 2500G) and serum (10 min, 4000G) samples were then centrifuged [7]. The samples were frozen at − 20 °C and then transferred to − 70 °C [7]. After the study the horses were returned to their owner.

### Analysis of serum and plasma sample

The analyses were carried out at the laboratory of the Department of Forensic Medicine of the Wroclaw Medical University.

### Chemicals and reagents

The following reagents were used: water (Chromasolv® LC–MS)^4^, acetonitrile (Chromasolv® LC–MS)^4^, methanol (Chromasolv® LC–MS)^4^, dichloromethane^4^, formic acid^4^, ammonium formate^5^; capsaicin^6^; dihydrocapsaicin^7^; phenacetin^8^. Stock solutions of capsaicin, dihydrocapsaicin and phenacetin were prepared in methanol at a concentration of 10 mg/mL. The standard solutions were stored in a freezer at − 20 °C.

### Instrumentation

Chromatographic analysis was performed using an ultra-high performance liquid chromatograph UHPLC Shimadzu Nexera X2^9^. The particles were separated using an ACQUITY UPLC® BEH C18^10^ 1.7 μm, 2.1 × 50 mm column with a thermostat at 40 °C. A mixture of 10 mL ammonium formate/0.1% formic acid in water (A) and 0.1% formic acid in acetonitrile (B) was used as a mobile phase. The gradient elution was carried out at constant flow 0.5 mL/min. The following gradient was applied: 0 min. - 5% B, 4 min. – 95% B, 4.5 min - 95% B, 5 min. – 5% B and then 7 min 5% B. Return to initial gradient compositions (95% A and 5% B) was performed at 5 min.

A triple-quadrupole mass spectrometer (QqQ, Shimadzu 8050)^11^ equipped with an ESI ion source, was used to detect the studied compounds. The MRM mode was employed to establish the presence of the studied substances. The analytes were then quantified by multiple reaction monitoring (MRM). MRM transitions were m/z 306.10 → 137.05; 94.10; 122.05 for capsaicin, m/z 308.40 → 137.10; 94.10; 122.10 for dihydrocapsaicin, and m/z 180.30 → 110.05; 93.10; 65.05 for phenacetin.

The retention times for capsaicin and dihydrocapsaicin were 3.2 and 3.4 min, respectively. Positive ionization was performed. The injected volume was 1 μL. The lower limit of quantification (LLOQ) for capsaicin and dihydrocapsaicin was 0.5 pg/mL and 1 pg/mL, respectively. The LLOQ was considered as the lowest calibration concentration with a relative standard deviation (%RSD) precision below 20%.

### Sample preparation

The sample (200 μL) was transferred into a 2-mL plastic tube, and 20 μL of an internal standard (Phenacetin 100 ng/mL) was added. A liquid-liquid extraction using dichloromethane (1 mL) was carried out for five minutes. The samples were then centrifuged at 10000 rpm. The organic phase (0.9 mL) was transferred into a 2-mL Eppendorf tube and evaporated to dryness under a stream of nitrogen (at 40 °C). The extract was dissolved in 25 μL of methanol, transferred to a glass insert and analysed using UHPLC-QqQ-MS/MS. Stock solutions were diluted with methanol to obtain working standard solutions at the following concentrations of capsaicin and dihydrocapsaicin: 5, 10, 100, 500, 1000, 5000 and 10,000 pg/mL. Calibration points were prepared by diluting the appropriate working solution with horse serum. The final concentrations of the calibrators were: 0.5, 1, 10, 50, 100, 500 and 1000 pg/mL horse serum for capsaicin and dihydrocapsaicin. QC samples (capsaicin and dihydrocapsaicin at 1 pg/mL) were run with each batch of samples.

## Results

**Fig. 1:**
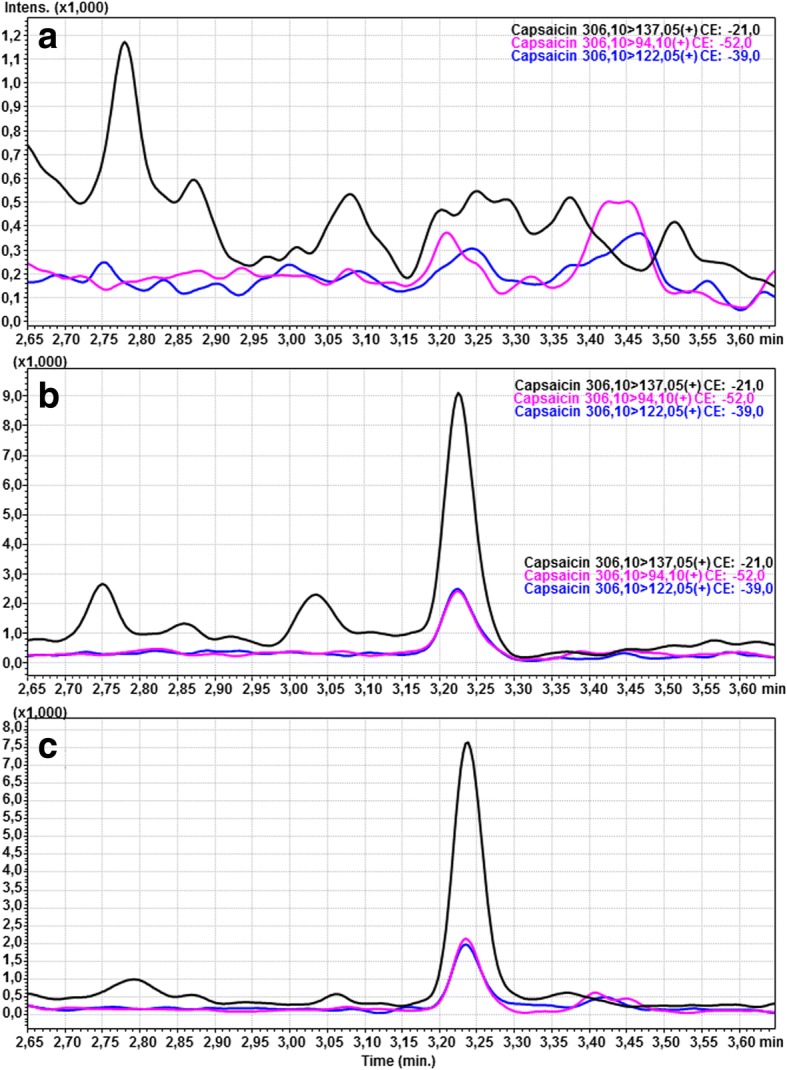
MRM chromatograms of capsaicin in the blank serum (**a**), spiked serum sample (at a concentration of 0.5 pg/mL) with capsaicin (**b**), case sample –horse number 9 (**c**)

None of the horses showed signs of discomfort, pain or stress associated with the effects of capsaicin. Detailed patient data are presented in Table 1. None of the results exceeded the lower limit of quantification (LLOQ), which amounted to 0.5 pg/mL for capsaicin and 1 pg/mL for dihydrocapsaicin. At T0, capsaicin was not detected in the blood serum or plasma. In two of the nine horses (22.22%), the concentration of capsaicin was quantifiable in the blood serum 12 h (T12) following the last gel administration although the obtained values were lower than the lower limit of quantification and amounted to 0.345 pg/mL and 0.135 pg/mL in horse no.4 and horse no.9, respectively (Fig. 1). Capsaicin was not detected in the blood serum more than 12 h following its last application. Capsaicin was not detected in the blood plasma samples. Dihydrocapsaicin was not detected in any of the samples.

**Table 1:** Detailed patient data

Number	Sex	Age	Breed	Weight [kg]
1.	gelding	18	german riding horse	500
2.	gelding	14	thoroughbred	517
3.	mare	17	halfbreed	506
4.	mare	14	pony	310
5.	mare	16	halfbreed	453
6.	mare	14	pony	300
7.	mare	15	halfbreed	447
8.	mare	10	arabian horse	440
9.	mare	6	arabian horse	452

## Discussion

Capsaicin is a strong and highly selective exogenous agonist of the transient receptor potential cation channel subfamily V member 1 (TRPV-1), also known as the vanilloid receptor 1 [8–10]. In literature, the local analgesic effect of capsaicin was reported to result from a depletion of substance P in nerve endings in the skin [11]. Currently, this mechanism is thought to play a minor analgesic role [12]. Topical administration of capsaicin decreases skin sensitivity and reduces the sensation of pain in a process known as “defunctionalisation” [13]. Defunctionalisation consists of a temporary loss of the membrane potential, an inability to transport neurotrophic factors and a reversible retraction of the nerve endings from the epidermis and skin [13–15]. The side effects of capsaicin use include a transient, local reaction that appears in the form of a pain, burning or erythema following the first administration [13]. Side effects, such as permanent skin denervation and nociceptive dysfunction, are theoretical and have not been described in clinical scenarios [16]. Higher concentrations of capsaicin (e.g. 10%) cause severe pain in horses [17].

In horses, capsaicin is considered a doping substance and is prohibited by the State of Pennsylvania Racing Commissions and FEI due to its strong analgesic effect and the duration of analgesia that extends beyond the time of the race/parkour [6]. Four of the 15 samples collected from show jumping horses at the Beijing Olympics in 2008 during anti-doping tests revealed the presence of capsaicin, causing those horses to be disqualified [7]. Gels containing capsaicin are widely available worldwide in equestrian shops. They are often used by sports and pleasure horses owners for mild lameness, or oedema as a first line treatment prior to a veterinary medical consultation. Such use is intended for curative purposes, including limiting pain and lameness, rather than doping. The described regimen for the use of the gel containing capsaicin, based on a five day gel administration together with polar bandages is the most common treatment scheme used by horse owners. The authors of this study marked capsaicin 12 h after its administration due to the fact that the study focused on detecting capsaicin administered for therapeutic use, not doping. You et al. 2013 studied the topical use of a 0.025% capsaicin gel and found that it reached its peak concentration two hours following its administration (241.9 pg/mL), and that it decreased to baseline values within 24 h (4.7 pg/mL) [7]. Those authors suggested that capsaicin is undetectable in blood plasma 48 h after application [7]. Hence, the purpose of this study was to determine the duration of capsaicin and dihydrocapsaicin detectability during long-term use and to assess whether capsaicin can be detected in blood serum and plasma.

The obtained results suggest a short-term detectability of capsaicin even after its long-term use. The results of this study did not exceed the LLOQ. However, the applied method enabled the authors to detect lower concentrations of the analytes although those concentrations did not meet the LLOQ. Hence, in two cases (horses no. 4 and 9), the chromatography of the studied sample at T12 had a similar analytical signal to the LOQ sample which signifies that capsaicin was present in the tested material at a very low concentration. The obtained results are contrary to those of You et al. who found that the concentration of capsaicin 24 h following its administration amounted to 4.7 pg/mL, despite the use a similar methodology and the same detection method in both studies [7]. In addition, dihydrocapsaicin was not detected in any of the samples, which stands in contrast to the findings of You et al., who noted detectable levels of dihydrocapsaicin 24 h after its administration [7]. The obtained results may be caused by breed differences between the studies, the different hair and feathers length as well as differences in the gel ingredients (especially the medium). In this study, isopropanol was the medium of the applied gel, which determined the best gel absorbance in humans [18]. Due to the possibility of local irritation caused by capsaicin, the application area was not shaved despite differing hair length among the studied horses. In addition, a polar bandage was applied to the treated area according to the treatment regimen, potentially causing some of the gel to absorb into the bandage material rather than the skin. Despite the availability of many capsaicin detection methods, such as: colorimetry/spectrophotometry, complexion chromatography, liquid chromatography (LC), gas chromatography and liquid chromatography tandem mass spectrometry (LC-MS) the authors chose to use ultra-high-performance liquid chromatography - tandem mass spectrometry, described in literature as fast, selectively sensitive, reproducible, reliable and fully validated [7, 19–25]. Future studies on the detectability of capsaicin, especially used for anti-doping testing, should consider assessing its metabolites for example vanillyamine and vanillic acid which are present invitro in the human skin [26].

The obtained results may be useful for horse owners and veterinary professionals to determine the period that should lapse from the administration of gels containing capsaicin (long-term therapeutic use) to the participation of the horses in riding events in accordance with the FEI [6]. Based on these results, capsaicin cannot be detected in horse serum 24 h after its administration using the therapeutic five day regimen and a commercially available gel together with a polar bandage. However, the absorbance of capsaicin (as well as its detectability) may be influenced by the type of hair at the application site, the size of the animal, the type of medium and numerous other factors. The second aim of the study was to determine the best study material for the detection of capsaicin and dihydrocapsaicin. According to the current study, serum is the best material for the detection of capsaicin. In the study by You et al. 2013, capsaicin and dihydrocapsaicin were measured just in blood plasma [7]. The studies of capsaicin metabolism have shown that only 0.095% of the unchanged circulating substance is excreted in urine and a small amount is excreted in faeces [27, 28]. Attempts have been made to determine the concentration of capsaicin in horse urine, but the tests have proven to be insensitive. They were found to detect capsaicin at a concentration of 10 ng/mL, indicating this method to be inadequate for routine horse evaluation [29]. The detection of capsaicin in horse hair has not been established so far, which should be the subject of further research. Horse hair from the mane and tail is usually used for the long-term evaluation of doping substances [30–32]. It provides a longer detection time than blood serum, plasma or urine, but it renders the determination of the time of substance intake impossible [30]. Current studies demonstrate the possibility to detect anabolic steroids, clenbuterol and other therapeutic substances not listed in the Prohibited Substances List in horse hair [30–32]. To date, the detectability of capsaicin in horse hair has not been determined. Hence, this should be studied further.

## Conclusion

After long-term (five days) use of capsaicin in horses in the form of a topical gel and a polar bandage, the substance is not detected in blood serum 24 h after the last gel application. The absorption and detection time of capsaicin in horses probably depends on the size and breed, hair length on the limbs, the kind of product and type of application.

## Abbreviations

FEI: Federation Equestrian International
LC: Liquid chromatography
LC-MS: Liquid chromatography tandem mass spectrometry
LLOQ: Lower limit of quantification
MRM: Multiple reaction monitoring
TRPV-1: Transient receptor potential cation channel subfamily V member 1
UHPLC: Ultra-high performance liquid chromatograph
UHPLC-QqQ-MS/MS: Ultra-high performance liquid chromatography coupled with a triple quadrupole mass spectrometry

## Footnotes

1 Sartadt, Germany

2 Ohaus Explorer, E1RR80, Ohaus, Switzerland

3 Horse Gel, Eclipse Biofarmab, Sweden

4 Sigma-Aldrich, Steinheim, Germany

5 Sigma-Aldrich, India

6 Sigma-Aldrich, Switzerland

7 Sigma-Aldrich, China

8 Aldrich Chemistry, China

9 Kyoto, Japan

10 Waters, Ireland

11 Kyoto, Japan

## References

[1] Cordell GA, Arajou OE (1993). Capsaicin: Identification, nomenclature, and pharmacotherapy. Ann Pharmacother.

[2] Hayman M, Kam PCA (2008). Capsaicin: a review of its pharmacology and clinical applications. Curr Anaesth Crit Care.

[3] Seino KK, Foreman JH, Greene SA, Goetz TE, Benson GJ (2003). Effects of topical Perineural capsaicin in a reversible model of equine foot lameness. J Vet Intern Med.

[4] Aley JP, Adams NJ, Ladyman RJ, Fraser DL (2015). The efficacy of capsaicin as an equine repellent for chewing wood. J Vet Behav.

[5] Govidarajan VS (1985). Capsicum production, technology, chemistry and quality. Part I: history, cultivation and primary processing. CRC Cr Rev Food Sci.

[6] 2017 Equine Prohibited Substances List FEI. https://inside.fei.org/sites/default/files/2018%20Equine%20Prohibited%20Substances%20List.pdf. Accessed 12 June, 2018.

[7] You Y, Uboh C, Soma LR, Guan F, Taylor D, Li X, Liu Y, Chen J (2013). Validated UHPLC–MS-MS method for rapid analysis of capsaicin and Dihydrocapsaicin in equine plasma for doping control. J Anal Toxicol.

[8] Alawi K, Keeble J (2010). The paradoxical role of the transient receptor potential vanilloid 1 receptor in inflammation. Pharmacol Ther.

[9] Caterina MJ, Schumacher MA, Tominaga M, Rosen TA, Levine JD, Julius D (1997). The capsaicin receptor: a heat-activated ion channel in the pain pathway. Nature.

[10] Conway SJ (2008). TRPing the switch on pain: an introduction to the chemistry and biology of capsaicin and TRPV1. Chem Soc Rev.

[11] Winters J, Bevan S, Campbell AE (1995). Capsaicin and pain mechanism. Brit J Anaesth.

[12] Hill R (2000). NK1 (substance P) receptor antagonists – why are they not analgesic in humans?. Trends Pharmacol Sci.

[13] Anand P, Bley K (2011). Topical capsaicin for pain management: therapeutic potential and mechanisms of action of the new high-concentration capsaicin 8% patch. Brit J Anaesth..

[14] Holzer P (2008). The pharmacological challenge to tame the transient receptor potential vanilloid-1 (TRPV1) nocisensor. Br J Pharmacol.

[15] Polydefkis M, Hauer P, Sheth S, Sirdofsky M, Griffin JW, McArthur JC (2004). The time course of epidermal nerve fibre regeneration: studies in normal controls and in people with diabetes, with and without neuropathy. Brain.

[16] Scholzen T, Armstrong CA, Bunnet NW, Luger TA, Olerud JE, Ansel JC (1998). Neuropeptides in the skin: interactions between the neuroendocrine and the skin immune systems. Exp Dermatol.

[17] Gleerup KB, Forkman B, Lindegaard C, Andersen PH (2015). An equine pain face. Vet Anaesth Analg.

[18] Pershing LK, Reilly CA, Crolett JL, Crouch DJ (2004). Effects of vehicle on the uptake and elimination kinetics of capsaicinoids in human skin in vivo. Toxicol Appl Pharm.

[19] Gibbs H, O’Garro LO (2004). Capsaicin content of West Indies hot pepper cultivars using colorimetric and chromatographic techniques. HortScience.

[20] Constant HL, Cordell GA, West DP, Johnson JH (1995). Separation and quantification of capsaicinoids using complexation chromatography. J Nat Prod.

[21] Cooper TH, Guzinski JA, Fisher C (1991). Improved high performance liquid chromatography method for the determination of major capsaicinoids in capsicum oleoresins. J Agr Food Chem.

[22] Karnka R, Rayanakorn M, Watanesk S, Vaneesorn Y (2002). Optimization of high-performance liquid chromatographic parameters for the determination of capsaicinoids compounds using the simple method. Anal Sci.

[23] Thomas BV, Schreibe AA, Weisskopf CP (1998). Simple method for quantitation of capsaicinoids in peppers using capillary gas chromatography. J Agr Food Chem.

[24] Kozukue N, Han JS, Kozukue E, Lee S, Kim LA, Lee KR, Levin CE, Friedman M (2005). Analysis of eight capsaicinoids in peppers and peppercontaining foods by high-performance liquid chromatography and liquid chromatography-mass spectrometry. J Agr Food Chem.

[25] Reilly CA, Crouch DJ, Yost GS, Fatah AA (2002). Determination of capsaicin, Nonivamide, and Dihydrocapsaicin in blood and tissue by liquid chromatography-tandem mass spectrometry. J Anal Toxicol.

[26] Chanda S, Bashir M, Babbar S, Koganti A, Bley K (2008). In vitro hepatic and skin metabolism of capsaicin. Drug Metab Dispos.

[27] Suresh D, Srinivasan K (2010). Tissue distribution and elimination of capsaicin, piperine and curcumin following oral intake in rats. Indian J Med Res.

[28] Reyes-Escogido ML, Gonzalez-Mondragon EG, Vazquez-Tzompantzi E (2011). Chemical and pharmacological aspects of capsaicin. Molecules.

[29] Stanley SMR, Wee WK, Lim BH, Foo HC (2007). Direct-injection screening for acidic drugs in plasma and neutral drugs in equine urine by differential-gradient LC–LC coupled MS/MS. J Chromatogr B.

[30] Anielski P (2008). Hair analysis of anabolic steroids in connection with doping control—results from horse samples. J Mass Spectrom.

[31] Dunnett M, Lees P (2004). Hair analysis as a novel investigative tool for the detection of historical drug use/misuse in the horse: a pilot study. Equine Vet J.

[32] Schlupp A, Anielski P, Thieme D, Mülle RK, Meyer H, Ellendorff F (2004). The β-agonist clenbuterol in mane and tail hair of horses. Equine Vet J.

